# Sharp constant of Hardy operators corresponding to general positive measures

**DOI:** 10.1186/s13660-018-1660-8

**Published:** 2018-04-10

**Authors:** Xudong Nie, Dunyan Yan

**Affiliations:** 1 Department of Mathematics and Physics, Shijiazhuang Tiedao University, Shijiazhuang, P.R. China; 2 School of Mathematical Sciences, University of the Chinese Academy of Sciences, Beijing, P.R. China

**Keywords:** 42B20, 42B35, Hardy operator, Best constant, Sharp problem

## Abstract

We investigate a new kind of Hardy operator $H_{\mu}$ with respect to arbitrary positive measures *μ* and prove that $H_{\mu}$ is bounded on $L^{p}(d\mu)$ with an upper constant $p/(p-1)$. Moreover, we characterize a sufficient condition about the measure which makes $p/(p-1)$ to be the $L^{p}$-norm of $H_{\mu}$.

## Introduction

Let *μ* be a positive measure on $[0,\infty)$ and *f* be a nonnegative *μ*-measurable function. Define Hardy operator with respect to the measure *μ* by

1 $$ H_{\mu}f(x)=\frac{1}{\mu([0,x])} \int_{[0,x]}f(t)\,d\mu(t), $$

if $0<\mu{([0,x])}<\infty$, and set $H_{\mu}f(x)=0$, if $\mu{([0,x])}=0$ or ∞.

Observe that if *μ* is Lebesgue measure, then $H_{\mu}$ becomes the classical Hardy operator

2 $$ Hf(x)=\frac{1}{x} \int_{0}^{x}f(t)\,dt, $$

and if $\mu=\sum_{k=1}^{\infty}\delta_{k}$, then $H_{\mu}$ becomes the discrete Hardy operator

$$\mathcal{H}f(k)=\frac{f(1)+\cdots+f(k)}{k}. $$

For $1< p<\infty$, reference [1] showed that the two operators are bounded on $L^{p}$ and $l^{p}$ respectively. Moreover, for both, the best constants are $p/(p-1)$ and the maximizing functions do not exist. We refer the reader to [2–6] for the background material and further references.

Hardy operator has a close relationship with Hardy–Littlewood maximal operator. From the point of rearrangement, *Hf* is equivalent to *Mf* (see reference [7]). In reference [8], Grafakos considered the $L^{p}$-boundedness for the maximal functions associated with general measures. In this paper, we shall discuss the sharp problems about $H_{\mu}$. We will show that the operator $H_{\mu}$ is bounded on $L^{p}(d\mu)$ with an upper bound no more than $p/(p-1)$. Furthermore, we will characterize a sufficient condition about *μ* such that $\Vert H_{\mu} \Vert _{L^{p}\rightarrow L^{p}}=p/(p-1)$.

From the definition about $H_{\mu}$, it is not necessary to consider the points *x* such that $\mu([0,x])=0$ or ∞. Therefore, we let

$$a=\inf\bigl\{ x:\mu\bigl([0,x]\bigr)>0\bigr\} , $$

and

$$b= \textstyle\begin{cases} \infty& \mbox{if } B= \emptyset,\\ \inf B& \mbox{if } B\neq\emptyset, \end{cases} $$

where *B* denotes the set $\{x:\mu([0,x])=\infty \mbox{ or } \mu([x,\infty))=0\}$. Then we call that the measure *μ* is supported in the interval $[a,b]$.

For the case of weak type inequality, the best constant from $L^{p}(d\mu)$ to $L^{p,\infty}(d\mu)$ is always 1.

### Theorem 1.1

*Let* *μ* *be a positive measure on* $[0,\infty]$ *and* $1\leq p<\infty$. *Then we have*

$$\Vert H_{\mu} \Vert _{L^{p}(d\mu)\rightarrow L^{p,\infty}(d\mu)}=1. $$

### Theorem 1.2

*Suppose that* *μ* *is supported in* $[a,b]$ *and* $f\in L^{p}(d\mu)$ *with* $1< p<\infty$. *For* $f\neq0$, *define*

$$\mathcal{R}_{\mu}(f)=\frac{ \Vert H_{\mu}f \Vert _{L^{p}(d\mu)}}{ \Vert f \Vert _{L^{p}(d\mu)}}. $$

*Then the following statements hold*:

(i) $\Vert H_{\mu}f \Vert _{L^{p}(d\mu)}\leq\frac{p}{p-1} \Vert f \Vert _{L^{p}(d\mu)}$ *holds for arbitrary positive measure* *μ*.
(ii) *There exists no function* *f* *such that* $\mathcal{R}_{\mu}(f)=\frac{p}{p-1}$ *holds*.

### Theorem 1.3

*If* *μ* *satisfies one of the following conditions*:

*Condition* 1. $\mu([a,b])=\infty$ *and*

$$\lim_{x\rightarrow b}\frac{\mu([a,x])}{\mu([a,x))}=1; $$

*Condition* 2. $\{a\}$ *is not an atom of* *μ*, *and*

$$\lim_{x\rightarrow a}\frac{\mu([a,x])}{\mu([a,x))}=1, $$

*then we have*

$$\sup_{f\in L^{p}(d\mu),f\neq0}\mathcal{R}_{\mu}(f)=\frac{p}{p-1}. $$

We remark that there indeed exist some measures so that

3 $$\begin{aligned} \sup_{f\in L^{p}(d\mu),f\neq0}\mathcal{R}_{\mu}(f)< \frac{p}{p-1}. \end{aligned}$$

For example, it is easy to know that the Dirac measure $\delta_{0}$ satisfies inequality (3). In this paper, we will give some more complex counterexamples.

## Preliminary and lemmas

In the study of sharp problems, the rearrangement of function is a very useful tool. Let

$$d_{f}(s)=\mu\bigl(\bigl\{ \vert f \vert >s\bigr\} \bigr). $$

Then the rearrangement of *f* is defined by

$$f^{*}(t)=\inf\bigl\{ s>0:d_{f}(s)\leq t\bigr\} . $$

By the properties of the rearrangement, we can easily have

$$\Vert f \Vert _{L^{p}(d\mu)}= \bigl\Vert f^{*} \bigr\Vert _{L^{p}(dm)}. $$

We refer the reader to [9] for more properties of rearrangement. In reference [1], Hardy gave the following result.

### Lemma 2.1

(G.H. Hardy and J.E. Littlewood)

*Let* $(X,\mu)$ *be a measurable space*. *If* $f, g\in\mathcal{M}(X,\mu)$, *then*

$$\int_{X} \vert fg \vert \,d\mu\leq \int_{0}^{\infty}f^{*}(t)g^{*}(t)\,dt $$

*holds*.

Moreover, the theory of rearrangement plays an important role in proving the existence of maximizing function. This is because of the following lemma introduced by Lieb [10].

### Lemma 2.2

*Suppose that* $(M, \Sigma, \mu)$ *and* $(M',\Sigma',\mu')$ *are two measure spaces*. *Let* *X* *and* *Y* *be* $L^{p}(M,\Sigma,\mu)$ *and* $L^{q}(M',\Sigma',\mu')$ *with* $1\leq p\leq q<\infty$. *Let* *A* *be a bounded linear operator from* *X* *to* *Y*. *For* $f\in X$ *with* $f\neq0$, *set*

$$\mathcal{R}(f)=\frac{ \Vert Af \Vert _{Y}}{ \Vert f \Vert _{X}} $$

*and*

$$N=\sup\bigl\{ \mathcal{R}(f):f\neq0\bigr\} . $$

*Let* $\{f_{j}\}$ *be a uniform norm*-*bounded maximizing sequence for* *N*, *and assume that* $f_{j}\rightarrow f\neq0$ *and that* $A(f_{j})\rightarrow A(f)$ *pointwise almost everywhere*. *Then* *f* *maximizes*, *i*.*e*., $\mathcal{R}(f)=N$.

## The boundedness of weak-$L^{p}$

In this section, we first prove Theorem 1.1. For the sake of clarity, we define a function as

$$F_{\mu}(x):=\mu\bigl([0,x]\bigr). $$

Obviously $F_{\mu}$ increases as $x\rightarrow\infty$. It follows from Lemma 2.1 and the definition of $H_{\mu}$ that

4 $$\begin{aligned} H_{\mu}f(x)&=\frac{1}{\mu([0,x])} \int_{[0,x]}f(t)\,d\mu(t) \\ &\leq \frac{1}{F_{\mu}(x)} \int_{[0,F_{\mu}(x)]}f^{*}(t)\,dt \\ &=Hf^{*} \bigl(F_{\mu}(x) \bigr). \end{aligned}$$

Let

$$E_{\mu}^{f^{*}}(\lambda):= \bigl\{ x:Hf^{*}\bigl(F_{\mu}(x) \bigr)>\lambda \bigr\} . $$

Note that $f^{*}$ decreases, so we easily have that $Hf^{*}$ decreases as well. If we take

$$x_{0}=\sup\bigl\{ x:Hf^{*}\bigl(F_{\mu}(x)\bigr)>\lambda\bigr\} , $$

then it implies that

$$E_{\mu}^{f^{*}}(\lambda)=[0,x_{0}). $$

Thus, we can obtain that

$$\bigl\{ x:Hf^{*}(x)>\lambda\bigr\} \supset\bigl[0,F_{\mu}(x_{0})\bigr). $$

We conclude that

5 $$ \mu \bigl(\bigl\{ x:Hf^{*}\bigl(F_{\mu}(x)\bigr)>\lambda \bigr\} \bigr)\leq F_{\mu}(x_{0}) \leq \bigl\vert \bigl\{ x:Hf^{*}(x)>\lambda\bigr\} \bigr\vert , $$

where $\vert \cdot \vert $ denotes the Lebesgue measure. It follows from inequalities (4) and (5) that

6 $$\begin{aligned} \frac{\sup_{\lambda>0}\lambda \mu(\{x:H_{\mu}f(x)>\lambda\})^{\frac{1}{p}}}{ \Vert f \Vert _{L^{p}(d\mu)}}&\leq \frac{\sup_{\lambda>0}\lambda \mu(\{x:Hf^{*}(F_{\mu}(x))>\lambda\})^{\frac{1}{p}}}{ \Vert f^{*} \Vert _{L^{p}(dm)}} \\ &\leq \frac{\sup_{\lambda>0}\lambda \vert \{x:Hf^{*}(x)>\lambda\} \vert ^{\frac{1}{p}}}{ \Vert f^{*} \Vert _{L^{p}(dm)}}. \end{aligned}$$

Since $f^{*}\in L^{p}(dm)$, by Hölder’s inequality, we have that

7 $$ Hf^{*}(x)=\frac{1}{x} \int_{0}^{x}f^{*}(t)\,dt\leq \biggl(\frac{1}{x} \int_{0}^{x} \bigl\vert f^{*}(t) \bigr\vert ^{p}\,dt \biggr)^{\frac{1}{p}} \leq x^{-\frac{1}{p}} \bigl\Vert f^{*} \bigr\Vert _{L^{p}(dm)}. $$

Thus it is obvious to obtain that

8 $$ \bigl\vert \bigl\{ x:Hf^{*}(x)>\lambda\bigr\} \bigr\vert \leq \bigl\vert \bigl\{ x:x^{-\frac{1}{p}} \bigl\Vert f^{*} \bigr\Vert _{L^{p}(dm)}>\lambda \bigr\} \bigr\vert = \frac{ \Vert f^{*} \Vert _{L^{p}(dm)}^{p}}{\lambda^{p}}. $$

From inequality (6) and inequality (8), we have

$$\frac{\sup_{\lambda>0}\lambda \mu(\{x:H_{\mu}f(x)>\lambda\})^{\frac{1}{p}}}{ \Vert f \Vert _{L^{p}(d\mu)}}\le1. $$

That is,

9 $$ \frac{ \Vert H_{\mu}f \Vert _{L^{p,\infty}(d\mu)}}{ \Vert f \Vert _{L^{p}(d\mu)}}\le1 $$

holds. This is equivalent to

10 $$ \Vert H_{\mu} \Vert _{L^{p}(d\mu)\rightarrow L^{p,\infty}(d\mu)}\leq1. $$

Next it suffices to show that the constant 1 is sharp for inequality (10).

Take $0\leq x_{1}< x_{2}<\infty$ such that $0<\mu ([x_{1},x_{2}] )<\infty$. Let $g=\chi_{[x_{1},x_{2}]}$. It is easy to obtain

$$\Vert H_{\mu}g \Vert _{L^{p,\infty}(d\mu)}= \Vert g \Vert _{L^{p}(d\mu)}. $$

The proof is completed.

## $L^{p}$-boundedness of the operator $H_{\mu}$ with upper bound $p/(p-1)$

Now we will show the results (i) and (ii) of Theorem 1.2.

### Proof

Following the proof of (5), we obtain

11 $$ \int_{[0,\infty]}\bigl(f\bigl(\mu\bigl([0,x]\bigr)\bigr) \bigr)^{p}\,d\mu(x)\leq \int_{[0,\infty]}f^{p}(x)\,dx. $$

By inequality (11), we conclude that

12 $$\begin{aligned} \Vert H_{\mu}f \Vert _{L^{p}(\mathbb {R}_{+},d\mu)}&= \biggl( \int_{\mathbb {R}_{+}} \biggl\vert \frac{1}{\mu([0,x])} \int_{[0,x]}f(t)\,d\mu(t) \biggr\vert ^{p}\,d\mu(x) \biggr)^{\frac{1}{p}} \\ &\leq \biggl( \int_{\mathbb {R}_{+}} \biggl\vert \frac{1}{F_{\mu}(x)} \int_{[0,F_{\mu}(x)]}f^{*}(t)\,dt \biggr\vert ^{p}\,d\mu (x) \biggr)^{\frac{1}{p}} \\ &= \biggl( \int_{\mathbb {R}_{+}} \bigl\vert Hf^{*}\bigl(F_{\mu}(x)\bigr) \bigr\vert ^{p}\,d\mu(x) \biggr)^{\frac{1}{p}} \\ &\leq \biggl( \int_{\mathbb {R}_{+}} \bigl\vert Hf^{*}(x) \bigr\vert ^{p}\,dx \biggr)^{\frac{1}{p}}. \end{aligned}$$

It follows from the inequality of classical Hardy operator that

13 $$ \biggl( \int_{\mathbb {R}_{+}} \bigl\vert Hf^{*}(x) \bigr\vert ^{p}\,dx \biggr)^{\frac{1}{p}} \leq\frac{p}{p-1} \bigl\Vert f^{*} \bigr\Vert _{L^{p} (dm)} =\frac{p}{p-1} \Vert f \Vert _{L^{p}(d\mu)}. $$

Combining inequality (12) with inequality (13), we have

$$\Vert H_{\mu}f \Vert _{L^{p}(\mathbb {R}_{+},d\mu)}\leq\frac{p}{p-1} \Vert f \Vert _{L^{p}(d\mu)}. $$

Since the sharp function for the classical Hardy operator does not exist, it is easy to know from inequality (12) that there exists no function *f* such that $\mathcal{R}_{\mu}(f)=\frac{p}{p-1}$. The proof of the result (ii) of Theorem 1.2 is completed. □

## A characterization of the measure *μ* which ensures $\sup_{f\neq0}\mathcal{R}_{\mu}(f)=p/(p-1)$

In this section, we try to characterize the measure *μ* which ensures $\sup_{f\neq0}\mathcal{R}_{\mu}(f)=p/(p-1)$. We regard *μ* as a complete atom measure by giving an appropriate partition on $[0,\infty]$. We first present a partition on $[0,\infty]$ by the following two lemmas.

### Lemma 5.1

*Let* *μ* *be a positive measure that is supported on* $[0,\infty]$. *If* $\mu([0,\infty])=\infty$ *and*

$$\lim_{x\rightarrow\infty}\frac{\mu(\{x\})}{\mu([0,x])}=0, $$

*then there exists a partition on* $[0,\infty]$ *as*

$$I_{0}=[0,x_{1}],\qquad I_{1}=(x_{1},x_{2}], \ldots,\qquad I_{k}=(x_{k},x_{k+1}], \ldots, $$

*such that*

$$\mu(I_{k+1})\geq\mu(I_{k}), $$

*and*

$$\lim_{k\rightarrow\infty}\frac{\mu({I_{k}})}{\mu([0,x_{k+1}])}=0. $$

### Proof

Let $x_{1}$ be any positive number. Denote $I_{0}=[0,x_{1}]$. Since *μ* is supported on $[0,\infty]$, we have

$$\mu(I_{0})>0. $$

For $k=2$, we let

$$x_{2}=\inf\bigl\{ x:\mu \bigl((x_{1},x] \bigr)\geq\mu \bigl([0,x_{1}]\bigr)\bigr\} . $$

For $k>2$, we let

$$x_{k}=\inf\bigl\{ x:\mu \bigl((x_{k-1},x] \bigr)\geq\mu \bigl((x_{k-2},x_{k-1}]\bigr)\bigr\} . $$

Denote $I_{k}=[x_{k-1},x_{k}]$ with $k=2,3,\ldots $ . Since $\mu([0,\infty])=\infty$, we easily have

$$\lim_{k\rightarrow\infty}x_{k}=\infty. $$

Thus, $\{I_{k}\}$ obviously constitutes a partition of $[0,\infty]$.

We first show that

$$\mu(I_{k})\geq\mu(I_{k-1}) $$

and

14 $$\begin{aligned} \mu\bigl((x_{k},x_{k+1})\bigr)\leq \mu(I_{k-1}). \end{aligned}$$

By our construction, for any $x>x_{k+1}$, it follows that

$$\mu\bigl((x_{k},x]\bigr)\geq\mu\bigl((I_{k-1})\bigr). $$

Thus the property of measure implies that

$$\mu(I_{k}) =\mathop{\lim_{x>x_{k+1}}}_{ x\rightarrow x_{k+1}}\mu\bigl((x_{k},x]\bigr)\geq \mu(I_{k-1}). $$

Moreover, if $x_{k}< x< x_{k+1}$, then $\mu([x_{k},x])<\mu(I_{k-1})$. Thus, it follows that

$$\mu\bigl((x_{k},x_{k+1})\bigr)=\mathop{\lim_{x< x_{k+1} }}_{ x\rightarrow x_{k+1}} \mu\bigl((x_{k},x]\bigr)\leq\mu(I_{k-1}). $$

To complete the proof, it remains to show that

$$\lim_{k\rightarrow\infty}\frac{\mu({I_{k-1}})}{\mu([0,x_{k}])}=0. $$

This is equivalent to prove that, for any $\epsilon>0$, there is an integer $N>0$ such that

$$\frac{\mu({I_{k-1}})}{\mu([0,x_{k}])}\leq2\epsilon $$

holds for $k\geq N$.

In order to prove this result, we divide the set $\mathbb {Z}^{+}\setminus\{1\}$ into two parts:

15 $$ F_{\epsilon} := \biggl\{ k\in\mathbb{Z}:k\geq2, \frac{\mu(\{x_{k}\})}{\mu ((x_{k-1},x_{k}))}< \epsilon \biggr\} $$

and

16 $$ G_{\epsilon}:= \biggl\{ k\in\mathbb{Z}:k\geq2, \frac{\mu(\{x_{k}\} )}{\mu((x_{k-1},x_{k}))}\geq\epsilon \biggr\} . $$

By definition (16), if $k\in G_{\epsilon}$, then we have

17 $$\begin{aligned} \mu(I_{k-1})\leq \biggl(1+\frac{1}{\epsilon} \biggr)\mu \bigl(\{x_{k}\}\bigr). \end{aligned}$$

We discuss the problem in two cases:

*Case* I. $G_{\epsilon}$ is not a finite set.

*Case* II. $G_{\epsilon}$ is a finite set.

If $G_{\epsilon}$ is not a finite set, then by equality $\lim_{x\rightarrow\infty}\frac{\mu(\{x\})}{\mu([0,x])}=0$, there exists an integer $N\in G_{\epsilon}$ such that, for any $k\geq N$,

18 $$\begin{aligned} \frac{\mu(\{x_{k}\})}{\mu([0,x_{k}])}< \frac{\epsilon^{2}}{1+\epsilon}. \end{aligned}$$

Thus if $k>N$ and $k\in G_{\epsilon}$, then by inequalities (17) and (18), we have

19 $$ \frac{\mu({I_{k-1}})}{\mu([0,x_{k}])}\leq\epsilon. $$

On the other hand, if $k> N$ and $k\in F_{\epsilon}$, since $G_{\epsilon}$ is not a finite integer and $N\in G_{\epsilon}$, we can find a series of integers $k_{0}$, $k_{0}+1$, …, *k*, such that $k_{0}\in G_{\epsilon}$, and

$$k_{0}+1,\ldots,k \in F_{\epsilon}. $$

By the definition of $F_{\epsilon}$ and inequality (14), we can conclude that if $i\in F_{\epsilon}$, then

20 $$\begin{aligned} \mu\bigl((x_{i-1},x_{i}]\bigr)&=\mu\bigl((x_{i-1},x_{i}) \bigr)+\mu\bigl(\{x_{i}\}\bigr) \\ &\leq(1+\epsilon)\mu\bigl((x_{i-1},x_{i})\bigr) \\ &\leq(1+\epsilon)\mu\bigl((x_{i-2},x_{i-1}]\bigr). \end{aligned}$$

It immediately implies from inequality (20) that

21 $$\begin{aligned} \mu\bigl((x_{k_{0}},x_{k}]\bigr)&=\sum _{i=k_{0}+1}^{k}\mu\bigl((x_{i-1},x_{i}]\bigr) \\ &\geq\sum_{i=k_{0}+1}^{k}(1+ \epsilon)^{i-k}\mu \bigl((x_{k-1},x_{k}]\bigr) \\ &=\mu\bigl((x_{k-1},x_{k}]\bigr)\frac{1- (\frac{1}{1+\epsilon} )^{k-k_{0}}}{1-\frac{1}{1+\epsilon}}. \end{aligned}$$

Thus, by inequality (21), we have

22 $$\begin{aligned} \frac{\mu({I_{k-1}})}{\mu([0,x_{k}])}&\leq\frac{\mu ((x_{k-1},x_{k}])}{\mu((x_{k_{0}-1},x_{k}])} \leq\frac{1-\frac{1}{1+\epsilon}}{1- (\frac{1}{1+\epsilon } )^{k-k_{0}}} \\ &\leq\frac{\epsilon}{1- (\frac{1}{1+\epsilon} )^{k-k_{0}}}. \end{aligned}$$

Since $k_{0}\in G_{\epsilon}$, inequalities (14) and (20) imply

23 $$ \frac{\mu({I_{k-1}})}{\mu([0,x_{k}])}\leq(1+\epsilon)^{k-k_{0}} \frac {\mu({I_{k_{0}-1}})}{\mu([0,x_{k}])}\leq(1+\epsilon)^{k-k_{0}}\epsilon. $$

If $(1+\epsilon)^{k-k_{0}}> 2$, by inequality (22), we have

$$\frac{\mu({I_{k-1}})}{\mu([0,x_{k}])}\leq2\epsilon. $$

If $(1+\epsilon)^{k-k_{0}}\leq2$, by inequality (23), we have

$$\frac{\mu({I_{k-1}})}{\mu([0,x_{k}])}\leq2\epsilon. $$

At last, we conclude that if $k>N$ and $k\in F_{\epsilon}$, then

24 $$ \frac{\mu({I_{k-1}})}{\mu([0,x_{k}])}\leq2\epsilon. $$

The proof of Case I is complete.

If $G_{\epsilon}$ is a finite set, then we can find an integer $k_{0}$ such that $k\in F_{\epsilon}$ for $k>k_{0}$. Then, by inequality (22), we can find a big enough integer *N* such that

$$\frac{\mu({I_{k-1}})}{\mu([0,x_{k}])}\leq2\epsilon $$

if $k\geq N$. The proof is completed. □

### Lemma 5.2

*Suppose that* *μ* *is supported in* $[0,\infty]$. *If* $\mu(\{0\})=0$ *and* $\lim_{x\rightarrow0}\frac{\mu([0,x])}{\mu([0,x))}=1$, *then there exists a partition on* $(0,1]$,

$$(x_{1},1], (x_{2},x_{1}], \ldots, (x_{k},x_{k-1}], \ldots, $$

*such that* $\lim_{k\rightarrow\infty}x_{k}=0$ *and*

$$\lim_{k\rightarrow\infty}\frac{\mu((x_{k},x_{k-1}])}{\mu([0,x_{k-1}])}=0. $$

### Proof

Without loss of generality, suppose

$$\mu\bigl([0,1]\bigr)=\sum_{k=1}^{\infty} \frac{1}{k^{2}}. $$

If $\mu(\{1\})<1$, then we set $k_{0}=0$. If $\mu(\{1\})\geq1$, then we set

$$k_{0}=\max \Biggl\{ m:\sum_{k=1}^{m} \frac{1}{k^{2}}\leq\mu\bigl(\{1\}\bigr) \Biggr\} . $$

It is easy to see that

$$\sum_{k=1}^{k_{0}+1}\frac{1}{k^{2}}>\mu\bigl( \{1\}\bigr). $$

Then we can find a positive real number $x_{1}<1$ such that

$$x_{1}=\sup \Biggl\{ x:\mu\bigl((x,1]\bigr)\geq\sum_{k=1}^{k_{0}+1} \frac{1}{k^{2}} \Biggr\} . $$

Proceeding in this way, we set

25 $$\begin{aligned} k_{i}=\max \Biggl\{ m:\sum_{k=1}^{m} \frac{1}{k^{2}}\leq\mu\bigl([x_{i},1]\bigr) \Biggr\} \end{aligned}$$

and

26 $$\begin{aligned} x_{i+1}=\sup \Biggl\{ x:\mu\bigl((x,1]\bigr)\geq\sum _{k=1}^{k_{i}+1}\frac{1}{k^{2}} \Biggr\} \end{aligned}$$

for $i\geq1$. By (27), (25), and (26), we can conclude

27 $$\begin{aligned} \sum_{k=1}^{k_{i}} \frac{1}{k^{2}}\leq\mu\bigl([x_{i},1]\bigr) \leq\mu\bigl((x_{i+1},1]\bigr) \leq\sum_{k=1}^{k_{i}+1}\frac{1}{k^{2}}\leq\mu \bigl([x_{i+1},1]\bigr). \end{aligned}$$

It is easy to see that $x_{i}>x_{i+1}$ and

$$\lim_{i\rightarrow\infty}\mu\bigl([x_{i},1]\bigr)\geq\lim _{i\rightarrow \infty}\sum_{k=1}^{k_{i}} \frac{1}{k^{2}}=\mu\bigl((0,1]\bigr). $$

Thus we have $\lim_{i\rightarrow\infty}x_{i}=0$. It is easy to see that

$$(x_{1},1], (x_{2},x_{1}], \ldots, (x_{k},x_{k-1}], \ldots, $$

divide $(0,1]$. It can be implied from inequality (27) that

28 $$ \mu\bigl([x_{i},1]\bigr)+\frac{1}{(k_{i}+1)^{2}}\geq\sum _{k=1}^{k_{i}+1}\frac{1}{k^{2}}\geq \mu\bigl((x_{i+1},1]\bigr). $$

To prove this partition satisfying the requirement of the lemma, we define two integer sets:

$$F_{\epsilon}= \biggl\{ k\geq1:\frac{\mu(\{x_{k}\})}{\mu ((x_{k+1},x_{k}])}< \epsilon \biggr\} $$

and

$$G_{\epsilon}= \biggl\{ k\geq1:\frac{\mu(\{x_{k}\})}{\mu ((x_{k+1},x_{k}])}\geq\epsilon \biggr\} , $$

where *ϵ* is an arbitrary positive real number. Since $\lim_{x\rightarrow0}\frac{\mu([0,x])}{\mu([0,x))}=1$, we have $\lim_{x\rightarrow0}\frac{\mu(\{x\})}{\mu([0,x))}=0$. It is easy to find an integer *N* such that

$$\frac{\mu(\{x_{i-1}\})}{\mu([0,x_{i-1}])}< 2\epsilon^{2} $$

for any integer $i>N$. Thus, by the construction of $G_{\epsilon}$, if $i>N$ and $i\in G_{\epsilon}$, we have

$$\frac{\mu((x_{i},x_{i-1}])}{\mu([0,x_{i-1}])}< 2\epsilon. $$

If $i\in F_{\epsilon}$, then we have

29 $$ \mu\bigl((x_{i+1},x_{i}]\bigr)\leq\frac{1}{1-\epsilon}\mu \bigl((x_{i+1},x_{i})\bigr). $$

By inequalities (28) and (29), we have

$$\begin{aligned} \frac{\mu((x_{i+1},x_{i}])}{\mu([0,x_{i}])}&\leq\frac{1}{1-\epsilon }\frac{\mu((x_{i+1},x_{i}))}{\mu([0,x_{i}])} \\ &= \frac{1}{1-\epsilon}\frac{\mu((x_{i+1},1])-\mu([x_{i},1])}{\mu ((0,1])-\mu((x_{i},1])} \\ &\leq \frac{1}{1-\epsilon}\frac{1/(k_{i}+1)^{2}}{\sum_{k=k_{i}+2}^{\infty}1/k^{2}}. \end{aligned}$$

Thus we can find a sufficiently large integer which is still denoted by *N* such that, for any integer $i>N$ and $i\in F_{\epsilon}$, there is

$$\frac{\mu((x_{i},x_{i-1}])}{\mu([0,x_{i-1}])}< 2\epsilon. $$

Since *ϵ* is an arbitrary real number, we have

$$\lim_{k\rightarrow\infty}\frac{\mu((x_{k},x_{k-1}])}{\mu([0,x_{k-1}])}=0. $$

The proof is completed. □

After finishing our preparations, we can give the proof of the result (iii) of the main theorem.

### Proof

Let

30 $$ { } T_{a,b}(x)=\textstyle\begin{cases} \tan (\frac{\pi}{2} (\frac{x-a}{b-a} ) ),& 0< b< \infty;\\ x-a,& b=\infty. \end{cases} $$

By equality (30), we can obtain a new measure denoted by $\mu_{T}$ which is supported in $[0,\infty]$ so that, for any open interval $(x,y)$, we have

$$\mu_{T}\bigl((x,y)\bigr)=\mu\bigl(\bigl(T_{a,b}^{-1}(x),T_{a,b}^{-1}(y) \bigr)\bigr). $$

Then it is easy to get

$$\sup\bigl\{ \mathcal{R}_{\mu}f \bigl\vert f\in L^{p}(d\mu) \bigr\} =\sup\bigl\{ \mathcal{R}_{\mu _{T}}f \bigr\vert f\in L^{p}(d \mu_{T})\bigr\} . $$

Thus it is enough to assume that the measure *μ* is supported in $[0,\infty]$.

We first consider Condition 1.

By Lemma 5.1, we can divide $\mathbb {R}^{+}$ into a series of intervals

$$[0,x_{1}],(x_{1},x_{2}], \ldots, (x_{k},x_{k+1}], \ldots, $$

such that

$$\lim_{k\rightarrow\infty}\frac{\mu((x_{k},x_{k+1}])}{\mu([0,x_{k}])}=0. $$

For any $\epsilon>0$, if we can find a function $f_{\epsilon}$ such that $\mathcal{R}(f_{\epsilon})\geq\frac{p}{p-1}-O(\epsilon)$, then the proof is completed.

By the property of the partition, there exists an integer *N* satisfying

$$\frac{\mu((x_{k},x_{k+1}])}{\mu([0,x_{k}])}< \epsilon $$

for $k\geq N$. This inequality is equivalent to

31 $$ \frac{\mu([0,x_{k+1}])}{\mu([0,x_{k}])}< 1+\epsilon. $$

Let

$$f_{\epsilon}=\sum_{k=N}^{\infty}\mu \bigl([0,x_{k+1}]\bigr)^{-\frac{1}{p}-\epsilon} \chi_{(x_{k},x_{k+1}]}. $$

First we estimate the norm of $f_{\epsilon}$

32 $$\begin{aligned} \Vert f_{\epsilon} \Vert _{L^{p}(d\mu)}&= \Biggl(\sum _{k=N}^{\infty}\mu \bigl([0,x_{k+1}] \bigr)^{-1-p\epsilon} \mu\bigl((x_{k},x_{k+1}]\bigr)\Biggr)^{\frac{1}{p}} \\ &\geq \biggl(\frac{1}{1+\epsilon} \biggr)^{\frac{1}{p}+\epsilon}\Biggl(\sum _{k=N}^{\infty}\mu\bigl([0,x_{k}] \bigr)^{-1-p\epsilon} \mu\bigl((x_{k},x_{k+1}]\bigr) \Biggr)^{\frac{1}{p}} \\ &= \biggl(\frac{1}{1+\epsilon} \biggr)^{\frac{1}{p}+\epsilon} \Biggl(\sum _{k=N}^{\infty} \int_{\mu([0,x_{k}])}^{\mu ([0,x_{k+1}])}\mu\bigl([0,x_{k}] \bigr)^{-1-p\epsilon}\,dt \Biggr)^{\frac{1}{p}} \\ &\geq \biggl(\frac{1}{1+\epsilon} \biggr)^{\frac{1}{p}+\epsilon} \biggl( \int_{\mu([0,x_{N}])}^{\infty} t^{-1-p\epsilon}\,dt \biggr)^{\frac{1}{p}} \\ &\geq \biggl(\frac{1}{1+\epsilon} \biggr)^{\frac{1}{p}+\epsilon} \biggl( \frac{1}{p\epsilon} \biggr)^{\frac{1}{p}} \mu\bigl([0,x_{N}] \bigr)^{-\epsilon}. \end{aligned}$$

Next, we estimate the value of $H_{\mu}f_{\epsilon}(x)$. When $k\geq N$ and $x_{k}< x\leq x_{k+1}$, we have

33 $$\begin{aligned} H_{\mu}f_{\epsilon}(x)&=\frac{1}{\mu([0,x])} \int_{[0,x]}f_{\epsilon }(t)\,d\mu(t) \\ &\geq\frac{1}{\mu([0,x_{k+1}])} \int_{[0,x_{k}]}f_{\epsilon}(t)\,d\mu (t) \\ &=\frac{1}{\mu([0,x_{k+1}])}\sum_{i=N}^{k-1}\mu \bigl([0,x_{i+1}]\bigr)^{-\frac{1}{p}-\epsilon} \mu\bigl((x_{i},x_{i+1}]\bigr) \\ &\geq \biggl(\frac{1}{1+\epsilon} \biggr)^{\frac{1}{p}+\epsilon}\frac{1}{\mu([0,x_{k+1}])} \sum _{i=N}^{k-1}\mu\bigl([0,x_{i}] \bigr)^{-\frac{1}{p}-\epsilon} \mu\bigl((x_{i},x_{i+1}]\bigr) \\ &= \biggl(\frac{1}{1+\epsilon} \biggr)^{\frac{1}{p}+\epsilon}\frac{1}{\mu([0,x_{k+1}])} \sum _{i=N}^{k-1} \int_{\mu([0,x_{i}])}^{\mu([0,x_{i+1}])} \mu\bigl([0,x_{i}] \bigr)^{-\frac{1}{p}-\epsilon}\,dt \\ &\geq \biggl(\frac{1}{1+\epsilon} \biggr)^{1+\frac{1}{p}+\epsilon }\frac{1}{\mu([0,x_{k}])} \int_{\mu([0,x_{N}])}^{\mu([0,x_{k}])}t^{-\frac{1}{p}-\epsilon }\,dt \\ &\geq \biggl(\frac{1}{1+\epsilon} \biggr)^{1+\frac{1}{p}+\epsilon }\frac{1}{1-\frac{1}{p}-\epsilon} \biggl(\mu\bigl([0,x_{k}]\bigr)^{-\frac{1}{p}-\epsilon}-\frac{\mu ([0,x_{N}])^{1-\frac{1}{p}-\epsilon}}{ \mu([0,x_{k}])} \biggr). \end{aligned}$$

Set

$$\begin{aligned} f_{\epsilon}^{(1)}&= \biggl(\frac{1}{1+\epsilon} \biggr)^{1+\frac{1}{p}+\epsilon}\frac {1}{1-\frac{1}{p}-\epsilon} \sum_{k=N}^{\infty} \mu\bigl([0,x_{k}]\bigr)^{-\frac{1}{p}-\epsilon}\chi _{(x_{k},x_{k+1}]} \\ &= \biggl(\frac{1}{1+\epsilon} \biggr)^{1+\frac{1}{p}+\epsilon}\frac {1}{1-\frac{1}{p}-\epsilon} f_{\epsilon} \end{aligned}$$

and

$$f_{\epsilon}^{(2)}= \biggl(\frac{1}{1+\epsilon} \biggr)^{1+\frac{1}{p}+\epsilon} \frac {1}{1-\frac{1}{p}-\epsilon} \sum_{k=N}^{\infty} \frac{\mu([0,x_{N}])^{1-\frac{1}{p}-\epsilon}}{\mu ([0,x_{k}])}\chi_{(x_{k},x_{k+1}]}. $$

Then we have

34 $$\begin{aligned} \bigl\Vert f_{\epsilon}^{(2)} \bigr\Vert _{L^{p}(d\mu)}&= \biggl(\frac{1}{1+\epsilon } \biggr)^{1+\frac{1}{p}+\epsilon} \frac{1}{1-\frac{1}{p}-\epsilon} \mu\bigl([0,x_{N}]\bigr)^{1-\frac{1}{p}-\epsilon} \Biggl(\sum _{k=N}^{\infty}\mu\bigl([0,x_{k}] \bigr)^{-p}\mu\bigl((x_{k},x_{k+1}]\bigr) \Biggr)^{\frac{1}{p}} \\ &\leq \biggl(\frac{1}{1+\epsilon} \biggr)^{\frac{1}{p}+\epsilon}\frac {1}{1-\frac{1}{p}-\epsilon} \mu \bigl([0,x_{N}]\bigr)^{1-\frac{1}{p}-\epsilon} \Biggl(\sum _{k=N}^{\infty}\mu\bigl([0,x_{k+1}] \bigr)^{-p}\mu \bigl((x_{k},x_{k+1}]\bigr) \Biggr)^{\frac{1}{p}} \\ &\leq \biggl(\frac{1}{1+\epsilon} \biggr)^{\frac{1}{p}+\epsilon}\frac {1}{1-\frac{1}{p}-\epsilon} \biggl(\frac{1}{p-1} \biggr)^{\frac{1}{p}} \mu\bigl([0,x_{N}] \bigr)^{-\epsilon}. \end{aligned}$$

By inequality (33), we have

$$\Vert H_{\mu}f_{\epsilon} \Vert _{L^{p}(d\mu)}\geq \bigl\Vert f_{\epsilon}^{(1)} \bigr\Vert _{L^{p}(d\mu)} - \bigl\Vert f_{\epsilon}^{(2)} \bigr\Vert _{L^{p}(d\mu)}. $$

From this result and inequalities (32) and (34), we can get

35 $$\begin{aligned} \mathcal{R}(f_{\epsilon}) &\geq\frac{ \Vert f_{\epsilon}^{(1)} \Vert _{L^{p}(d\mu)} - \Vert f_{\epsilon}^{(2)} \Vert _{L^{p}(d\mu)}}{ \Vert f_{\epsilon} \Vert _{L^{p}(d\mu )}} \\ &= \biggl(\frac{1}{1+\epsilon} \biggr)^{1+\frac{1}{p}+\epsilon} \frac{1}{1-\frac{1}{p}-\epsilon}- \frac{ \Vert f_{\epsilon}^{(2)} \Vert _{L^{p}(d\mu)}}{ \Vert f_{\epsilon} \Vert _{L^{p}(d\mu)}} \\ &\geq \biggl(\frac{1}{1+\epsilon} \biggr)^{1+\frac{1}{p}+\epsilon} \frac{1}{1-\frac{1}{p}-\epsilon}- \frac{ (\frac{1}{1+\epsilon} )^{\frac{1}{p}+\epsilon}\frac {1}{1-\frac{1}{p}-\epsilon} (\frac{1}{p-1} )^{\frac{1}{p}}}{ (\frac{1}{1+\epsilon } )^{\frac{1}{p}+\epsilon} (\frac{1}{p\epsilon} )^{\frac{1}{p}}}. \end{aligned}$$

Since *ϵ* is arbitrary, it is easy to imply $\sup_{f\neq 0}\mathcal{R}(f)=\frac{p}{p-1}$.

To prove condition (ii), by Lemma 5.2, we can part the intervals $(0,1]$ to

$$(x_{1},1], (x_{2},x_{1}], \ldots, (x_{k+1},x_{k}], \ldots $$

such that

$$\lim_{k\rightarrow\infty}\frac{\mu((x_{k+1},x_{k}])}{\mu((0,x_{k}])}=0. $$

Then, for any $\epsilon>0$, there is a sufficiently large integer *N* such that

$$\frac{\mu((x_{k+1},x_{k}])}{\mu((0,x_{k}])}< \epsilon $$

for $k\geq N$.

Thus, we have

$$\frac{\mu((0,x_{k+1}])}{\mu((0,x_{k}])}\geq1-\epsilon. $$

Let $f_{\epsilon}=\sum_{k=N}^{\infty}\mu((0,x_{k}])^{-\frac {1}{p}+\epsilon}\chi_{(x_{k+1},x_{k}]}$. Then, for $x_{k+1}< x\leq x_{k}$ and $k\geq N$, we have

36 $$\begin{aligned} H_{\mu}f_{\epsilon}(x)&=\frac{1}{\mu((0,x])} \int_{(0,x]}f_{\epsilon }(t)\,d\mu(t) \\ &\geq \frac{1}{\mu((0,x_{k}])} \int _{(0,x_{k+1}]}f_{\epsilon}(t)\,d\mu(t) \\ &=\frac{1}{\mu((0,x_{k}])}\sum_{i=k+1}^{\infty} \mu\bigl((0,x_{i}]\bigr)^{-\frac{1}{p}+\epsilon} \mu\bigl((x_{i+1},x_{i}]\bigr) \\ &\geq\frac{1}{\mu((0,x_{k}])}\frac{\mu((0,x_{k+1}])^{1-\frac{1}{p}+\epsilon}}{1-\frac{1}{p}+\epsilon} \\ &\geq\frac{(1-\epsilon)^{1-\frac{1}{p}+\epsilon}}{1-\frac{1}{p}+\epsilon }\mu\bigl((0,x_{k}]\bigr)^{-\frac{1}{p}+\epsilon} \end{aligned}$$

37 $$\begin{aligned} &=\frac{(1-\epsilon)^{1-\frac{1}{p}+\epsilon}}{1-\frac{1}{p}+\epsilon }f_{\epsilon}(x). \end{aligned}$$

It follows from inequality (36) that

$$\mathcal{R}(f_{\epsilon})\geq\frac{(1-\epsilon)^{1-\frac{1}{p}+\epsilon}}{1-\frac{1}{p}+\epsilon}. $$

Because *ϵ* is arbitrary, it is easy to know $\sup_{f}\mathcal {R}(f)\geq\frac{p}{p-1}$. The proof of the suffice part of Theorem 1.2 is then completed. □

## Counterexample

In this section we give some counterexamples that make $\sup_{f\neq 0,f\in L^{p}}\mathcal{R}(f)< p/(p-1)$. The following two lemmas tell us that we can limit our discussion to a special function set.

### Lemma 6.1

*Suppose* *μ* *is a positive measure on* $\mathbb {R}_{+}$ *and it has an atom* $x_{0}$. *If* $\{f_{n}\}$, $n=1,2,\ldots$ , *is a series of functions satisfying* $f_{n}(x_{0})=1$ *and*

$$\lim_{n\rightarrow\infty}\mathcal{R}_{\mu}(f_{n})= \frac{p}{p-1}, $$

*then we have*

$$\lim_{n\rightarrow\infty} \Vert f_{n} \Vert _{L^{p}(d\mu)}= \infty. $$

### Proof

Without loss of generality, we assume that $\mu(\{x_{0}\})=1$. If the assertion does not hold, then we can assume that there exists a constant *C* satisfying $\Vert f_{n} \Vert _{L^{p}(d\mu)}\leq C$. Let $f_{n}^{*}$ be the decreasing rearrangement of $f_{n}$, then it is easy to get $\Vert f_{n}^{*} \Vert _{L^{p}(dm)}\leq C$ and $f_{n}^{*}(1)\geq1$. Thus we have $f^{*}(x)\geq1$ for $0< x\leq1$. By Helly’s theorem, we can assume $\lim_{n\rightarrow\infty} f^{*}_{n}=f^{*}$ almost everywhere. Since $f_{n}^{*}$ is decreasing, we have

$$C^{p}\geq \bigl\Vert f_{n}^{*} \bigr\Vert _{L^{p}(dm)}^{p}\geq \int_{[0,R]} \bigl\vert f_{n}^{*}(t) \bigr\vert ^{p}\,dt\geq R \bigl\vert f_{n}^{*}(R) \bigr\vert ^{p}, $$

which is equivalent to $f_{n}^{*}(R)\leq CR^{-\frac{1}{p}}$. Thus, by the control convergence theorem,

38 $$ \lim_{n\rightarrow\infty}Hf_{n}^{*}(x)= \lim _{n\rightarrow\infty}\frac{1}{x} \int_{[0,x]}f_{n}^{*}(t)\,dt=Hf^{*}(x). $$

However, by inequality (12), we have

$$\mathcal{R}_{m}\bigl(f_{n}^{*}\bigr)\geq \mathcal{R}_{\mu}(f_{n}), $$

it obviously shows that $\{f_{n}^{*}\}$ is a maximizing sequence for *H*, i.e.,

$$\lim_{n\rightarrow\infty}\mathcal{R}\bigl(f_{n}^{*}\bigr)= \frac{p}{p-1}. $$

By $\lim_{n\rightarrow\infty} f^{*}_{n}=f^{*}$ and equality (38), using Lemma 2.2, we get $\mathcal{R}_{m}(f^{*})=\frac{p}{p-1}$, which contradicts the result about Hardy operator we have known. The proof is completed. □

### Lemma 6.2

*Suppose* *μ* *is a positive measure on* $\mathbb {R}_{+}$ *and it has an atom* $x_{0}$. *If*

$$\sup\bigl\{ \mathcal{R}_{\mu}f| f\in L^{p}(d\mu)\bigr\} = \frac{p}{p-1}, $$

*then there exists a series of functions* $\{f_{k}\}$, $k=1,2,\ldots$ , *and* $f_{k}(x_{0})=0$ *such that*

$$\lim_{k\rightarrow\infty}\mathcal{R}_{\mu}(f_{k})= \frac{p}{p-1}. $$

### Proof

It is obvious that we can assume there exists a series of functions $g_{k}$, $g_{k}(x_{0})=1$, such that

$$\lim_{k\rightarrow\infty}\mathcal{R}_{\mu}(g_{k})= \frac{p}{p-1}. $$

Let

$$f_{k}(x)=\textstyle\begin{cases} g_{k}(x),&x\neq x_{0},\\ 0,&x=x_{0}. \end{cases} $$

Then we have

39 $$ H_{\mu}f_{k}(x)=\textstyle\begin{cases} H_{\mu}g_{k}(x),&x< x_{0};\\ H_{\mu}g_{k}(x)-\mu(\{x_{0}\})/\mu([0,x]),&x\geq x_{0}. \end{cases} $$

By equality (39), we can get

40 $$ \bigl\Vert H_{\mu}(f_{k}) \bigr\Vert _{L^{p}}\geq \bigl\Vert H_{\mu}(g_{k}) \bigr\Vert _{L^{p}}- \biggl\Vert \frac{\mu(\{x_{0}\})}{\mu([0,\cdot])}\chi_{[x_{0},\infty ]} \biggr\Vert _{L^{p}}. $$

On the other hand, it is easy to obtain

41 $$ \Vert f_{k} \Vert _{L^{p}}\leq \Vert g_{k} \Vert +\mu\bigl(\{x_{0}\}\bigr)^{\frac{1}{p}}. $$

By Lemma 6.1, we know that $\lim_{k\rightarrow\infty} \Vert g_{k} \Vert _{L^{p}(d\mu)}=\infty$ and $\lim_{k\rightarrow\infty}\mathcal{R}_{\mu}(g_{k})=p/(p-1)$. By this result, together with inequalities (40) and (41), we can have

$$\lim_{k\rightarrow\infty}\mathcal{R}_{\mu}(f_{k})= \frac{p}{p-1}. $$

 □

Now we can give some counterexamples.

### Example 6.3

Suppose that *μ* is supported in $[a,b)$, $\mu(\{a\})>0$, and $\mu (\mathbb {R}_{+})<\infty$. Then $\sup_{f\neq0}\mathcal{R}_{\mu}(f)< p/(p-1)$.

### Proof

Suppose that the result is not valid. By Lemma 6.2, we can find a series of functions $\{f_{k}\}$, $f_{k}(a)=0$, such that

$$\lim_{k\rightarrow\infty}\mathcal{R}_{\mu}(f_{k})= \frac{p}{p-1}. $$

Let $A=\mu(\{a\})$, $B=\mu(\mathbb {R}_{+})$, and $\mu_{1}=\mu-A\delta_{a}$. Then we have

42 $$ H_{\mu}f_{k}(x)=\frac{\mu_{1}([0,x])}{\mu([0,x])} \frac{1}{\mu_{1}([0,x])} \int_{[0,x]}f_{k}\,d\mu_{1}\leq \frac{B-A}{B}H_{\mu_{1}}f_{k}(x) $$

and

43 $$ \Vert f_{k} \Vert _{L^{p}(d\mu)}= \Vert f_{k} \Vert _{L^{p}(d\mu_{1})}. $$

By inequalities (42) and (43), we obtain

$$\mathcal{R}_{\mu}(f_{k})\leq\frac{B-A}{B} \mathcal{R}_{\mu_{1}}(f_{k}) \leq\frac{B-A}{B} \frac{p}{p-1}. $$

It contradicts with $\lim_{k\rightarrow\infty}\mathcal{R}_{\mu }(f_{k})=p/(p-1)$. Then the counterexample is valid. □

### Example 6.4

If $\mu=\sum_{k=-\infty}^{\infty}\lambda^{k} \delta_{\lambda^{k}}$ with $\lambda>1$, then $\sup_{f\in L^{p}(d\mu)}\mathcal{R}f<\frac{p}{p-1}$.

### Proof

By the definition of *μ*, we have

44 $$\begin{aligned} H_{\mu}f(\lambda_{k})&= \frac{1}{\mu([0,\lambda_{k}])} \int _{[0,\lambda_{k}]}f(t)\,d\mu(t) \\ &=\frac{(\lambda-1)\sum_{i=-\infty}^{k}\lambda^{i} f(\lambda ^{i})}{\lambda^{k+1}} \\ &=\frac{\lambda-1}{\lambda}\sum_{i=-\infty}^{0} \lambda ^{i}f\bigl(\lambda^{i+k}\bigr) \end{aligned}$$

and

45 $$\begin{aligned} \bigl\Vert f\bigl(\lambda^{i}\cdot\bigr) \bigr\Vert _{L^{p}(d\mu)} &= \Biggl(\sum_{k=-\infty}^{\infty} \bigl\vert f\bigl(\lambda^{i+k}\bigr) \bigr\vert ^{p} \lambda ^{k} \Biggr)^{\frac{1}{p}} \\ &= \Biggl(\sum_{k=-\infty}^{\infty} \bigl\vert f\bigl(\lambda^{k}\bigr) \bigr\vert ^{p}\lambda ^{k-i} \Biggr)^{\frac{1}{p}} \\ &=\lambda^{-\frac{i}{p}} \Vert f \Vert _{L^{p}(d\mu)}. \end{aligned}$$

By inequalities (44), (45), and Minkowski’s inequality, it follows

46 $$\begin{aligned} \Vert H_{\mu}f \Vert _{L^{p}(d\mu)}&= \Biggl\Vert \frac{\lambda-1}{\lambda}\sum_{i=-\infty}^{0}\lambda ^{i}f\bigl(\lambda^{i}\cdot\bigr) \Biggr\Vert _{L^{p}(d\mu)} \\ &\leq \frac{\lambda-1}{\lambda}\sum_{i=-\infty}^{0} \lambda^{i} \bigl\Vert f\bigl(\lambda^{i}\cdot\bigr) \bigr\Vert _{L^{p}(d\mu)} \\ &=\frac{\lambda-1}{\lambda}\sum_{i=-\infty}^{0} \lambda^{i-\frac{i}{p}} \Vert f \Vert _{L^{p}(d\mu)} \\ &=\frac{\lambda-1}{\lambda-\lambda^{\frac{1}{p}}} \Vert f \Vert _{L^{p}(d\mu)}. \end{aligned}$$

It is easy to get $\frac{\lambda-1}{\lambda-\lambda^{\frac{1}{p}}}<\frac{p}{p-1}$. The proof is completed. □
